# An UV-photo and ionic dual responsive interpenetrating network hydrogel with shape memory and self-healing properties†

**DOI:** 10.1039/d2ra00619g

**Published:** 2022-05-18

**Authors:** Ziyi Li, Jiwei Cai, Miaohan Wei, Juncheng Chen

**Affiliations:** The First Dongguan Affiliated Hospital of Guangdong Medical University, The Second Clinical Medical College, Guangdong Medical University Dongguan 523808 China liziyi@gdmu.edu.cn

## Abstract

Shape memory hydrogels have attracted extensive attention in fields such as artificial tissues, biomimetic devices and diagnostics, and intelligent biosensors. However, the practical applications were hindered by the absence of self-healing capability and multi-stimuli-responsiveness. To address these issues, we developed a shape memory hydrogel with self-healing and dual stimuli-response performance. The hydrogel system was constructed *via* an interpenetrating network consisting of *in situ* radical polymerization and host–guest interaction. The hydrogel exhibited rapid self-healing property, which can be stretched after self-healing for 1 min at 25 °C. Besides, the hydrogel displayed varied swelling performance in different light or solvent conditions. Moreover, the hydrogel showed a dual stimuli-responsive shape memory effect to ultraviolet (UV) light and ionic strength in 1 min. Such a shape memory hydrogel with self-healing ability and multi-stimuli-responsive properties will offer an option toward intelligent soft materials for biomedical and bionic research.

## Abstract 2

A developed interpenetrating network hydrogel displayed self-healing performance and dual stimuli responsive shape memory upon ionic and ultraviolet light (UV) stimulation.

## Graphical abstract

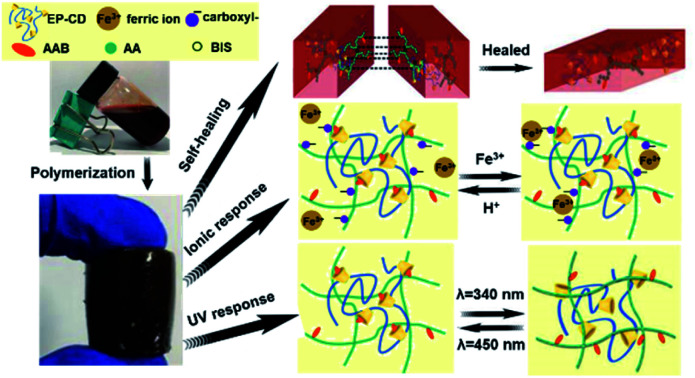

## Introduction

Shape memory hydrogels (SMHs) are a kind of soft material with a permanent crosslinked network for recovering to their original shapes and a molecular switch for fixing a temporary shape.^1,2^ Diverse interactions have been utilized in hydrogel networks to expand SMHs through reversible formation-destruction mechanisms,^1^ such as ionic coordination,^3–5^ hydrogen bonds,^6–9^ host–guest interactions,^10,11^ metal–ligand coordination,^12,13^ boronate ester bonds^14,15^ and Schiff base bonds.^13,16^ Meanwhile, SMHs are typical intelligent materials, which respond to various external stimuli such as heat, pH, light and ionic strength for controllable and reversible deformation.^1^ In recent years, SMHs have attracted plenty of attention in intelligent valves, soft robots and biomedicine.^17^ However, SMHs with a single function are far from satisfactory for wider potential applications duo to single stimulus-induced shape memory and lack of self-healing property.^2^ Hence, it is attractive to develop SMHs with functions such as multi-stimuli-response property, and self-healing behavior.

Introduction of different stimuli in one material contributed to developing multi-shape memory hydrogel and satisfy the practical application under different conditions. SMHs response to several stimuli may be achieved when two types of temporary cross-links are incorporated in the polymer network that respond to independent stimuli.^2^ Rapid diffusion of small molecules or switch in molecular structure could serve as triggers for shape memory. For example, complexation of carboxyl groups and ferric (III) ions provide the opportunity for fixing a temporary shape as an alternative to crystallizable domains and cleaving molecular switches by complexing agents under different pH condition.^17,18^ In addition, SMHs hydrogel with self-healing performance have attracted increasing research interest. Improving the mechanical properties contributed to expand the potential of the intelligent devise or artificial muscles in load-bearing applications.^19^ Moreover, enhancing self-healing property is also of great significance to restore their functionalities of SMHs after damage in real service life, which is an important index for materials.^2^ Many efforts have been adopted to develop self-healing hydrogels based on dynamic covalent bonding and non-covalent interaction. The self-healing mechanism includes imine bonds,^20,21^ boronate bonds,^22–24^ disulfide bonds,^25–27^ ionic interaction,^28^ hydrophobic interaction,^29^ hydrogen bonds^30,31^ and host–guest interaction.^32^ Among this, the hydrogels crosslinked from non-covalent interactions such as hydrogen bond and host–guest interaction are generally highly flexible and self-healing while those obtained from covalent bonding are highly stable.^33^ Among this, cyclodextrin (CD) and adamantine (Ad) are typical molecule for forming a host–guest complex with self-healing property.^34^

In this paper, a dual stimuli-responsive hydrogel with self-healing performance was fabricated by *in situ* radical reaction of acrylic acid (AA) and host–guest interaction between 4-acrylamide azobenzene (AAB) and polycyclodextrin (EP-CD). The AAB with *trans* configuration formed host–guest effect with EP-CD under 450 nm light. The carboxylic group along AA produced reversible complexation with ferric ions (Fe^3+^). Thus, the host–guest interaction and ionic complexation provided fixing force and achieved UV-photo and ionic dual response. Moreover, the abundant hydroxyl and carboxyl group in the hydrogel benefits forming hydrogen bond. The resultant hydrogel exhibited good self-healing performance because of the synergistic effect of hydrogen bond and host–guest interaction. Hence, this study may provide a strategy for the design and fabrication of dual-stimuli responsive hydrogel with shape memory and self-healing performance.

## Experimental

### Materials

Epichlorohydrin (99%), β-cyclodextrin (96%), aniline (98%) was received from energy-chemical Co., Ltd. Acrylic acid (98%) was supplied by TCI chemical Co., Ltd. Furanidine and sodium hydroxide were provided by xilong scientific Co., Ltd. The purity of the above reagents was analytical grade. Hydrogen chloride (37%) was supplied by xilong scientific Co., Ltd. All the other reagents were used as received.

### Synthesis of 4-acrylamide azobenzene (AAB)

Aniline (2.0 g, 21.7 mmol) was added dropwise in hydrochloric acid (37%, 6.5 mL), and diazotized at 0 to 5 °C with sodium nitrite solution (1.5 g, 21.7 mmol). The mixture was stirred for 1 h. A yellow transparent diazonium salt solution was obtained. Then, aniline (2.0 g, 21.7 mmol) and hydrochloric acid (1 mol L^−1^, 22.0 mL) were stirred vigorously at 0 to 5 °C to prepare the coupling solution. Diazonium salt solution was dropwise into the coupling solution at 0 to 5 °C. The system was stirred at 5 °C for 3 h. The final solution was slowly added to ammonia solution (1 mol L^−1^, 30.0 mL) to obtain yellow-orange precipitate of azo compound. The precipitate was filtered and washed with bicarbonate (pH = 8). The sediment was collected by filtration. Then, it was cleaned with deionized water and dried under vacuum. The resulting orange crystal was recrystallized from ethanol and named as 4-azoanilin.

At 4 °C, 4-azoaniline (3.0 g, 15.2 mmol) and triethylamine (3.0 mL, 8.5 mmol) were dissolved in 110.0 mL tetrahydrofuran. Then, acryloyl chloride (1.4 g, 15.0 mmol) dissolved in 10.0 mL tetrahydrofuran was added dropwise to the above mixed solution, and the mixed solution was stirred in an ice-water bath for 4 h. The precipitate was removed by filtration. Then, the crude product was purified by recrystallization with dichloromethane three times. Finally, the product was dried under vacuum.

### Synthesis of polycyclodextrin (EP-CD)

Weighed sodium hydroxide was dissolved in deionized water to prepare 15.0 wt% aqueous solution (2.6 g, 66.0 mmol). Then, β-cyclodextrin (5.0 g, 4.4 mmol) was added and stirred at room temperature for 3 h. Epichlorohydrin (4.1 g, 44.1 mmol) was added dropwise to the above solution. The mixture was continue stirred for 3 h. Subsequently, the reacted solution was added dropwise into acetone. After that, the supernatant was removed. Then, the supernatant was dissolved in deionized water and neutralized (pH = 7) with diluted hydrochloric acid solution. The solution was dialyzed in a dialysis bag with a molecular weight cut-off of 7000 Da, and the dialyzed solution was freeze-dried and product was obtained.

### Preparation of interpenetrating network hydrogel

The XY of AA_*x*_AAB_*y*_CD_*y*_ hydrogel meant multiple of concentration of acrylic acid (AA) and 4-acrylamide azobenzene (AAB) and polycyclodextrin (EP-CD). For example, AA_1_AAB_1_CD_1_ meant the concentration of AA, AAB and EP-CD in the hydrogel system was 1, 0.02 and 0.02 mol L^−1^, respectively. The preparation process was as follows: 1.5 g EP-CD, 0.08 g BIS, and 19.5 mL water was added in a three-necked flask, which was placed in an oil bath at 60 °C. Nitrogen was introduced to remove oxygen for 30 min. Then, it was moved into strip mold and sealed for oxygen removal. 0.1 g 4-acrylamide azobenzene (AAB) was weighed and dissolved in 3.6 g acrylic acid (AA). The mixed solution was deoxidized for 10 min and 0.2 g initiator potassium peroxodisulfate (KPS) was added. The mixed solution was injected into the mold with a needle tube. After sealed, the mold was placed in an oven at 60 °C and heated for 4 h to prepare hydrogel.

### Chemical structure characterization

The chemical structure was tested by fourier transform infrared spectrometer (FTIR, Prestige-21, Shimadzu Corporation of Japan). Among this, 4-aminoazobenzene, β-cyclodextrin and polycyclodextrin powder was tested by KBr tabletting method. The samples were fully dried, then mixed with potassium bromide powder according to the ratio of about 1 : 100, and fully ground, respectively. Then, the transmission data was tested with a certain test range (400–4000 cm^−1^) and the scanning times (16 times). Similarly, the resultant hydrogel was tested by reflection method with the test range (400 ∼ 4000 cm^−1^) and the scanning times (32 times).

### Thermal performance test

Thermal performance was evaluated by differential scanning calorimetry (DSC) test (DSC 6220, Japan Seiko) In the tests, 5∼10 mg xerogel was used. The temperature was raised from 20 °C to 200 °C for the first time at a rate of 10 °C min^−1^, and kept at 200 °C for 1 min. Then, it was cooled to 0 °C at a rate of 10 °C min^−1^. The second heating was 0 ∼ 200 °C with heating rate of 10 °C min^−1^.

### Mechanics performance test

The tensile properties were tested on electronic universal material testing machine (MDEL, Sansi Zongheng, Shenzhen, China). The samples presented rectangular with a size of 5 × 1 × 50 mm (width × thickness × length). The tensile rate was 20 mm min^−1^ at room temperature. The strain was defined as strain=(*l*/*l*_0_)×100%, where *l* was the stretching length and *l*_0_ was the initial length. Tensile stress was defined as tensile force applied over cross-sectional area. Stress was calculated with the equation: Stress = *F*/*S*_0_, where *F* was tensile force and *S*_0_ was initial cross-sectional area. During the test, silicone oil should be coated on the surface of hydrogel to prevent water evaporation.

### Self-healing performance test

Firstly, macroscopic self-healing property of hydrogel was evaluated. The hydrogel was made as rectangular with a size of 5 × 1 × 50 mm (width × thickness × length). Subsequently, it was cut with a knife. Then, it was connected for self-healing at 25 °C and its tensile condition was observed after 1 min. Besides, its self-healing ability was evaluated by hung a weight after the hydrogel self-healed for 30 min in humid environment (60 °C). Furthermore, self-healing capacity was quantitative analyzed by the universal material testing machine at 25 °C. After the samples were self-healed under different conditions, the tensile properties were tested. The definitions of strain and stress were the same as those in tensile properties. Self-healing rate was defined as *η* = *σ*_c_1__/*σ*c__0__, *σ*_c_1__ was the tensile strength of the initial sample, and *σ*_c_0__ was the tensile strength of the repaired sample.

### Swelling performance test

Swelling property of xerogels with different AA content or EP-CD content was tested by swelling for 24 h in deionized water at 25 °C. Then, swelling property of AA_2_AAB_1_CD_1_ xerogels was tested in different solvents at 25 °C. The samples swelled in deionized water, 0.01 mol L^−1^ 4-(phenylazo)benzoic acid (AzoCOOH) solution and 0.01 mol L^−1^ β-cyclodextrin (β-CD) solution, respectively. The tests were performed for 90 min at room temperature in the dark. Besides, swelling performance was evaluated under different illumination environments. The xerogel swelled in deionized water at room temperature for 90 min in ultraviolet light with wavelength of 340 nm, blue light with wavelength of 450 nm or a dark room, respectively. The swelling ratio of hydrogel is defined as *Q* = [(*W*_t_ − *W*_dry_)/*W*_dry_] × 100%, where *W*_t_ and *W*_dry_ was the weight of swollen hydrogel at predetermined time and the constant weight dried in vacuum drying oven, respectively. The solvent was changed when the swelling time exceeded 1 h.

### Shape memory performance test

The shape memory properties of hydrogels were tested under different environment at 25 °C. The ionic responsive shape memory performance was evaluated by putting hydrogel samples in Fe^3+^/H^+^ solution and observing their shape. The UV light responsive property was evaluated by recording the change of shape under 450 nm/340 nm illumination.

### Statistical analysis

Each of the experiments was repeated at least three times, and the values were expressed as means ± standard deviations.

## Results and discussion

### Chemical structure analysis

**Fig. 1:**
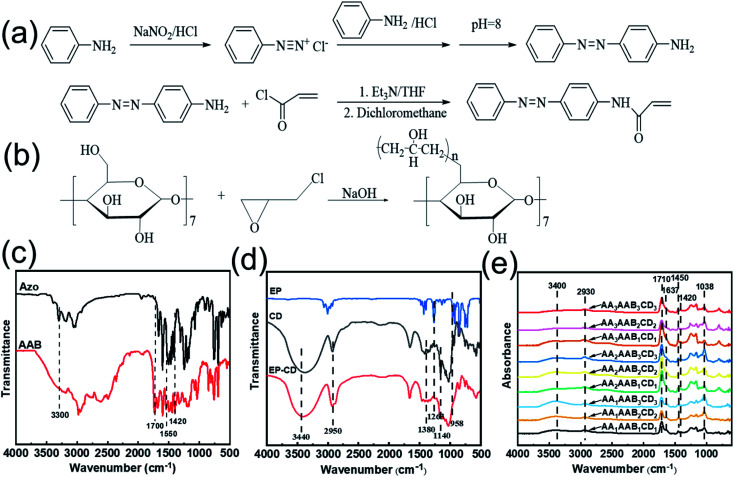
Synthesis route of AAB (a) and EP-CD (b), Fourier infrared transform spectroscopy characterization of AAB (c), EP-CD (d) and the hydrogel with different component (e).

4-Acrylamide Azobenzene (AAB) was synthesized according to the previous reports.^35,36^ The synthetic route of AAB was shown in Fig. 1a. FTIR spectrum of AAB was analyzed (Fig. 1c). The peak at 3300 cm^−1^ was assigned to stretching vibration of N–H bond in aromatic amine. And it disappeared in AAB. The characteristic peak was observed at 1700 cm^−1^, suggesting the stretching vibration of C=C bond. The peak at 1559 cm^−1^ was assigned to stretching vibration of N–H bond of secondary amide bond. The characteristic peak at 1420 cm^−1^ appeared, indicating stretching vibration of N=N bond in azobenzene. Therefore, AAB containing carbon–carbon double bond was successfully synthesized. Polycyclodextrin (EP-CD) was prepared by reaction between cyclodextrin and epichlorohydrin in alkaline environment^37^ (Fig. 1b). In FTIR spectrum of EP-CD (Fig. 1d), the peaks at 2950 cm^−1^ and 1380 cm^−1^ were ascribed as stretching vibration and variable angle vibration of C–H bond in β-cyclodextrin, respectively. The characteristic peak was observed at 3440 cm^−1^, indicating stretching vibration of O–H bond in β-cyclodextrin. The antisymmetric and symmetric stretching vibration of C–O–C bond in epichlorohydrin were at 958 and 1263 cm^−1^, respectively, which disappeared in EP-CD. Moreover, the stretching vibration of C–O–C bond at 1140 cm^−1^ was obviously weakened, suggesting that EP-CD was successfully synthesized.

The interpenetrating network hydrogel formed by free radical polymerization process and host–guest interaction. The free radical polymerization was promoted between the double bond of AA and AAB. The host–guest interaction was formed by the reaction between azobenzene group on AAB and cyclodextrin groups of EP-CD. The chemical structure of interpenetrating network hydrogel (AA_*x*_AAB_*y*_CD_*y*_) was analyzed by infrared spectrum (Fig. 1e). The wide absorption band of 3400 cm^−1^ is the stretching peak of liquid water. The stretching vibration and variable angle vibration of C–H bond in β-cyclodextrin appeared at 2930 cm^−1^ and 1450 cm^−1^, respectively.^38^ The peak at 1710 cm^−1^ suggested stretching vibration of C=O bond in acrylic acid. The peak at 1637 cm^−1^ was assigned as the extensional vibration of C=O bond in secondary amide. The characteristic peak at 1420 cm^−1^ and 1038 cm^−1^ indicated the stretching vibration of N=N bond in azo monomer and the stretching vibration of O–H bond in β-cyclodextrin, respectively.

### Preparation of interpenetrating network hydrogel

**Fig. 2:**
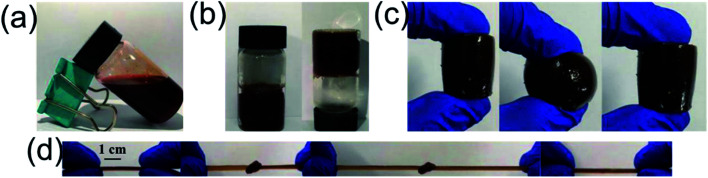
Gelation performance by inverted vial method and the macro analysis compression and tensile properties. The state before gelation (a) and after gelation (b), the test for compression and recoverability (c), and the test for knotting and tensile and recoverability (d).

Gelation performance was observed by inverted vial method.^39^ The precursor solution displayed sol performance and flowability before gelation (Fig. 2a). Once gelation, that resultant hydrogel couldn't flow in the bottle when the vial was inverted (Fig. 2b). The resultant cylindrical AA_3_AAB_2_CD_2_ hydrogel could be compressed and recoverable (Fig. 2c), suggesting the hydrogel was tough and recoverable. Moreover, the hydrogel with the length of 3 cm was prepared. It could be knotted and stretched to 10 cm (Fig. 2d). After the knot was untied, the hydrogel recovered to its original appearance. The results showed that the interpenetrating network hydrogel has excellent toughness.

### Thermal performance analysis

The thermal properties of the hydrogel were characterized by DSC (Fig. S1†), the glass transition temperature (*T*_g_) of polyacrylic acid was about 100 °C.^40^ However, *T*_g_ of the AA_0.5_AAB_1_CD_1_ hydrogel decreased to 46.1 °C. It might result from that the introduction of the cyclodextrin network greatly increased the molecular flexibility of the hydrogels. Furthermore, *T*_g_ increased from 46.1 to 60.7 °C with the increase of AA concentration from 0.5 mol L^−1^ to 2 mol L^−1^ (AA_0.5_AAB_1_CD_1_ to AA_2_AAB_1_CD_1_). When the AA content was kept a constant, *T*_g_ was almost the same. The results indicated that PAA network contributed to stronger intermolecular force and thermostability.

### Mechanical property analysis

**Fig. 3:**
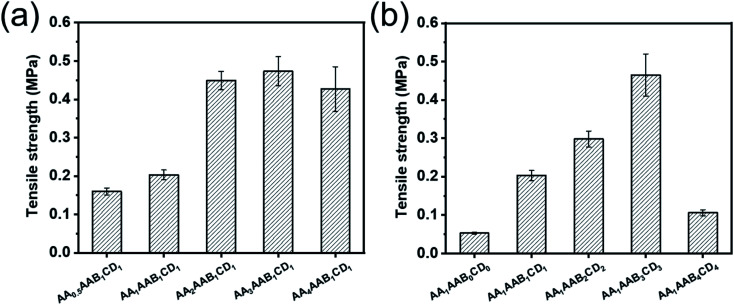
Evaluation of tensile property. Influence of AA content (a) and EP-CD content (b) on the tensile strength of hydrogels.

The mechanical properties of the hydrogels were further evaluated by universal tensile testing machine. The effect of AA or EP-CD content to the tensile strength of hydrogels was analyzed (Fig. 3). As shown in Fig. 3a, the tensile strength increased from 0.160 MPa to 0.473 MPa when the concentration of AA increased from 0.5 to 3 mol L^−1^ (AA_0.5_AAB_1_CD_1_ to AA_3_AAB_1_CD_1_). It could be explained that a large number of hydrogen bonds formed between carboxyl groups on polyacrylic acid and cyclodextrin with increase of AA content. When AA content was further increased to 4 mol L^−1^ (AA_4_AAB_1_CD_1_), the mechanical properties of hydrogel decreased slightly because molecular chain entanglement increased the viscosity of the system.^41,42^ Besides, when the concentration of EP-CD in precursor solution increased from 0 to 0.06 mol L^−1^ (AA_1_AAB_0_CD_0_ to AA_1_AAB_3_CD_3_), tensile strength of the resultant hydrogel increased from 0.053 MPa to 0.465 MPa (Fig. 3b). In this stage, the intermolecular force was increased and the mechanical property was improved. However, the tensile strength greatly decreased when the concentration was further increased to 0.08 mol L^−1^ (AA_1_AAB_4_CD_4_), indicating that excessive intermolecular aggregation in polycyclodextrin network affected the mechanical properties.^43^

### Evaluation of self-healing performance

**Fig. 4:**
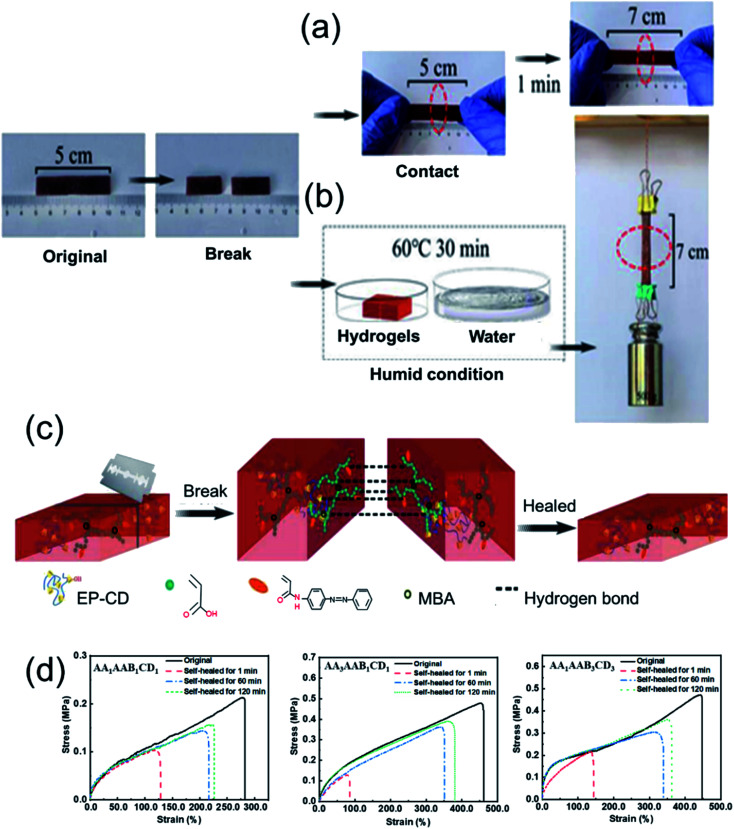
Evaluation of self-healing performance. The macroscopic self-healing performance of hydrogels after self-healing for 1 min at room temperature (a) or 30 min in a humid environment at 60 °C (b). (c) Diagram of self-healing mechanism. (d) The tensile strength of AA_1_AAB_1_CD_1_, AA_3_AAB_1_CD_1_ and AA_1_AAB_3_CD_3_ hydrogels and those after the corresponding hydrogels self-healing for 1, 60, 120 min.

The self-healable performance was evaluated by macroscopic observation. The hydrogel was cut off and connected together. After self-healing for 1 min at 25 °C, the hydrogel could be stretched (Fig. 4a). If the hydrogel was placed in a humid environment at 60 °C for self-healing for 30 min, it could bear a weight of 500 g and stretched (Fig. 4b), demonstrating the hydrogel has macroscopical self-healing property. Self-healing mechanism was shown in Fig. 4c. After the hydrogel was broken or damaged, hydrogen bonds contributed to the self-healing property,^22,44^ which could be formed between hydroxyl groups on EP-CD, carboxyl groups on PAA and amide groups on PAAB. Besides, the host–guest interaction as reversible bond was formed between EP-CD and PAAB when the sections were put together again. Hence, the self-healing capacity of the hydrogel was performed by hydrogen bonds and the host–guest interaction.

Self-healable property of the hydrogel was further demonstrated by quantitative analysis of tensile property. The stress–strain curves displayed tensile property of the original hydrogels with different components and the corresponding self-healing ones for 1, 60 or 120 min at room temperature in wet environment and the self-healing efficacy was further calculated (Fig. 4d and S2†). To prevent evaporation of water, the tests were performed in humid environment. The self-healing effect was improved when prolonging the self-healing time. In addition, the self-healable efficiency increased from 73% to 82% when AA content increased from 1 to 3 mol L^−1^ (AA_1_AAB_1_CD_1_ to AA_3_AAB_1_CD_1_) after the hydrogels self-healing for 120 min at 60 °C. It might be that a large number of hydrogen bonds provided by polyacrylic acid network structure formed higher physical cross-linking density after the hydrogel was fully self-healed. With the content of EP-CD in the hydrogel increasing from 0.02 to 0.06 mol L^−1^ (AA_1_AAB_1_CD_1_ to AA_1_AAB_3_CD_3_), the self-healing efficiency of EP-CD at 60 °C for 120 min decreased from 73% to 61% and then increased to 76%. At the beginning, the higher molecular cross-linking density leaded to uneven distribution of polycyclodextrins among chains. Thus, the self-healing effect was reduced. When the EP-CD content was further increased, the dispersion effect was further improved. Thus, the formed host–guest intermolecular between cyclodextrin along EP-CD and azo group on AAB contributed to repair the damage.^45–47^

### Swelling performance analysis

**Fig. 5:**
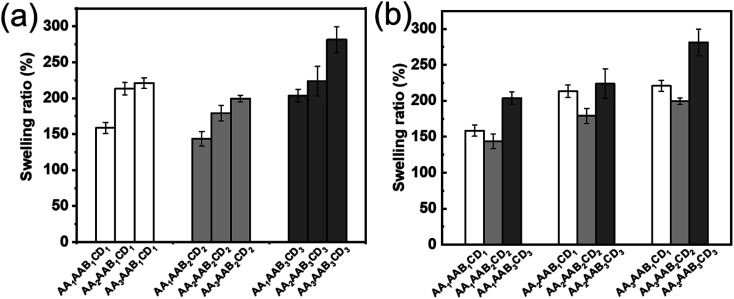
Swelling rate of hydrogels after swelling for 24 h. (a) The influence of AA content on swelling ratio of hydrogels with fixed EP-CD content. (b) The influence of EP-CD content on swelling ratio of hydrogels with fixed AA content.

The influence of AA and EP-CD components on the swelling performance was analyzed. The swelling ratio increased with AA content (Fig. 5a) at the fixed EP-CD content. The reason might be that carboxyl and hydrophilic on AA polymer chain contributed to quickly bound water.^48,49^ The influence of EP-CD content on the network structure of hydrogels was more complex (Fig. 5b). Initially, the increase of EP-CD content from 0.02 mol L^−1^ (AA_*x*_AAB_1_CD_1_) to 0.04 mol L^−1^ (AA_*x*_AAB_2_CD_2_) resulted to the decrease of swelling ratio, which might duo to entanglement and uneven distribution among molecular segments reduced the water storage capacity of hydrogels. When EP-CD content exceeded a certain amount (0.06 mol L^−1^, AA_*x*_AAB_3_CD_3_), swelling ratio was increased. It could be explained that a large number of hydroxyl groups in EP-CD could combine with water. Hence, it displayed good water storage capacity.

**Fig. 6:**
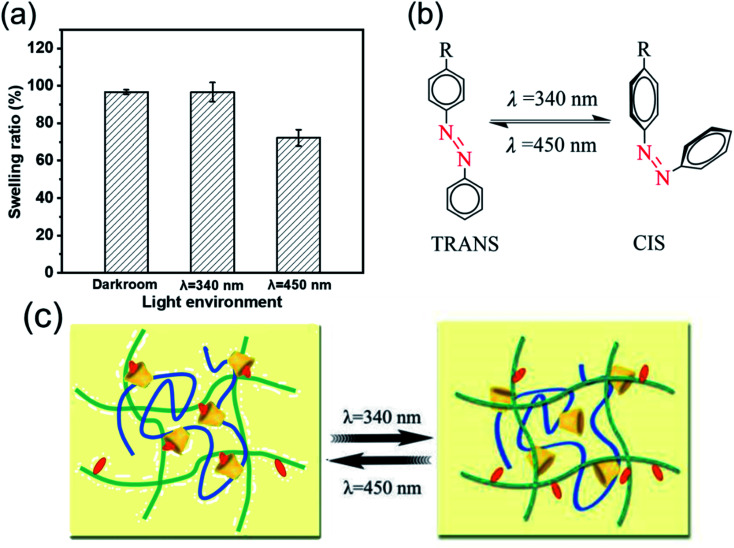
Swelling properties of AA_2_AAB_1_CD_1_ hydrogels under different wavelengths of light. (a) The quantitative analysis of swelling rate under different wavelengths of light. The mechanism affects the principle of swelling properties by *cis*–*trans* isomerization of AAB under different light (b) and the crosslinking of the host–guest effect (c).

To evaluate the potential of stimulus-response behavior by the host–guest effect, swelling performance of AA_2_AAB_1_CD_1_hydrogels under different light treatment or solvent conditions was further studied. The swelling ratio of samples treated at dark room or 340 nm light for 90 min was almost equal (97%) while those treated at 450 nm for 90 min reached to 72% (Fig. 6a), which demonstrated that AAB in the interpenetrating network hydrogels contributed to changing the crosslinking density and forming host–guest effect with EP-CD under 450 nm light. All the results could be explained with *cis*–trans isomerization of AAB under different light (Fig. 6b and 6c). The AAB produced *cis*-structure after treated with 340 nm light and showed *trans*-structure after receiving light at 450 nm.^50,51^ Compared with *cis*-structure, *trans*-structure at 450 nm was less sterically hindered, forming subject-object effect with EP-CD. Therefore, crosslinking density was improved and swelling ratio was decreased under 450 nm light.

**Fig. 7:**
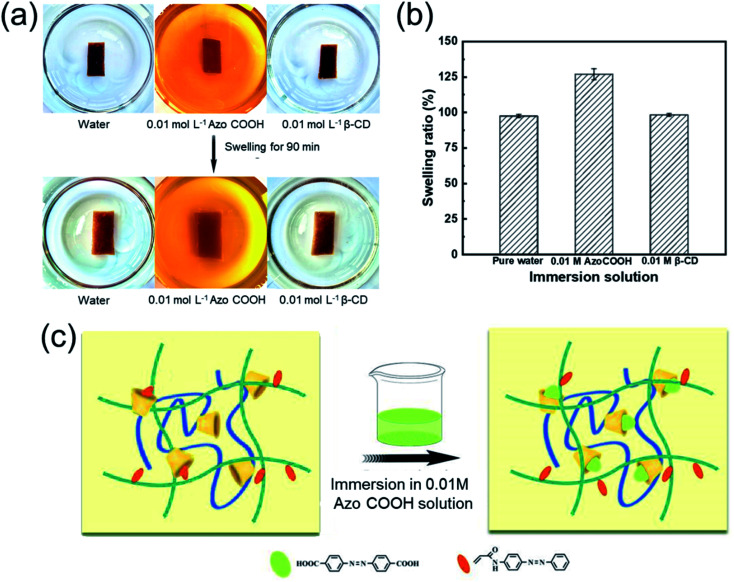
Swelling properties of AA_2_AAB_1_CD_1_ hydrogel in different solutions. (a) Macroscopic evaluation of the swelling performance. (b) Quantitative analysis of swelling rate in different solutions. (c) Influencing mechanism of Azo COOH solution on swelling properties.

Moreover, the AA_2_AAB_1_CD_1_ hydrogel displayed different swelling performance in varied solvents (Fig. 7). After the hydrogels were immersed in deionized water or 0.01 mol L^−1^ β-cyclodextrin for 90 min, the *Q* value reached almost to 98%. In contrast, the *Q* value increased to 127% after the hydrogels swelled in 0.01 mol L^−1^ AzoCOOH for 90 min. It could be explained that the bond capacity between AzoCOOH and cyclodextrin in EP-CD was larger than that of PAAB because the AzoCOOH as small molecule compound easily destroyed the original cross-linking structure and increased the *Q* value. The results demonstrated the reversible host–guest crosslinking existed in the hydrogel.

### Shape memory performance analysis

**Fig. 8:**
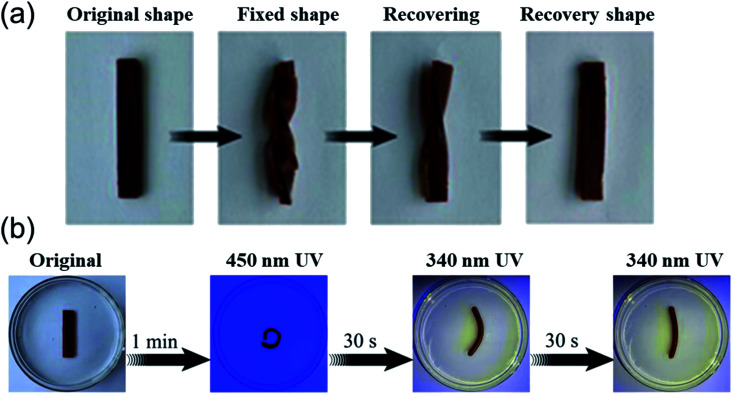
Stimulus-responsive of AA_2_AAB_1_CD_1_ hydrogels in 0.1 mol L^−1^ Fe^3+^/HCl solution. (a) Ionic response and (b) UV responsive shape memory of AA_2_AAB_1_CD_1_ hydrogel under 340 or 450 nm.

Furthermore, the shape memory performance of the AA_2_AAB_1_CD_1_ hydrogel was evaluated by Fe^3+^/HCl solution and UV light response. On the one hand, the original hydrogel was put into 0.1 mol L^−1^ Fe^3+^/HCl solution to bend and fix its shape, the color of the hydrogel became lighter, suggesting Fe^3+^ entered the hydrogel network. After the hydrogel with fixed shape was immersed in 0.1 mol L^−1^ HCl solution for 1 min, it displayed recoverability and completely recovered to its shape in 5 min (Fig. 8a). In the hydrogel system, carboxyl groups on acrylic acid could form ionic bonds with Fe^3+^, and the ionic bonds could be destroyed due to protonation of carboxyl groups in acidic condition, thus forming a dynamic controllable structure and shape memory effect.^18^ On the other hand, the shape of the original hydrogel could be fixed in 1 min under the illumination of 450 nm wavelength, and it started to recover once the illumination of 340 nm wavelength and the shape recovered after 1 min (Fig. 8b). It resulted from that azobenzene moiety on AAB in hydrogels was reversible *trans*–*cis* photo-isomerized.^52^*Cis*-structure of azobenzene moiety on AAB at ultraviolet light (340 nm) was more difficult to form host–guest effect with cyclodextrin along EP-CD compared with its *trans*-structure at visible light (450 nm).^53,54^ The reason was the same as that in swelling test. Therefore, the performance difference of this combination was served as photo-responsive shape memory effect.

## Conclusion

In this study, an interpenetrating network hydrogel with dual stimuli responsive shape memory and self-healing property was developed by *in situ* polymerization method based on free radical polymerization of PAA-co-PAAB and host–guest crosslinking of PAAB and EP-CD. Thermal performance analysis displayed the PAA network contributed to thermostability of the resultant hydrogel. Besides, hydrogen bonds among PAA and host–guest reaction endowed the resultant hydrogel with self-healing capacity, which was demonstrated by macroscopic observation and quantitative analysis of tensile property. Additionally, the components, light and solvent exhibited an influence on swelling property of the hydrogel. Moreover, the resultant hydrogel displayed dual responsive shape memory performance, which completely recovered to its shape in 5 min or 1 min in 0.1 mol L^−1^ HCl solution or the illumination of 340 nm wavelength, respectively. The smart shape memory hydrogel has potential applications in smart devices including self-healable electronic devices, soft robot and sensors.

## Conflicts of interest

There are no conflicts to declare.

## Supplementary Material

RA-012-D2RA00619G-s001
