# Supplementary material for: An UV-photo and ionic dual responsive interpenetrating network hydrogel with shape memory and self-healing properties
Source: RSC Adv. 2022 May 18;12(24):15105–14. doi: 10.1039/d2ra00619g (PMC9116958; doi:10.1039/d2ra00619g)
Supplement: RA-012-D2RA00619G-s001 [file RA-012-D2RA00619G-s001.pdf]

Electronic Supplementary Material (ESI) for RSC Advances.
This journal is © The Royal Society of Chemistry 2022

Supporting information

An UV-photo and ionic dual responsive interpenetrating network hydrogel with shape memory and self-healing properties

Ziyi Li,* Jiwei Cai, Miaohan Wei, Juncheng Chen

The First Dongguan Affiliated Hospital of Guangdong Medical University, The Second Clinical Medical College, Guangdong Medical University, Dongguan, 523808, China

Corresponding author: E-mail: liziyi@gdmu.edu.cn

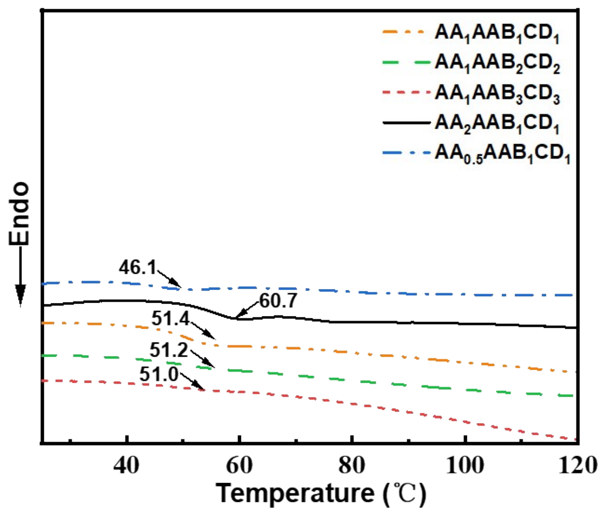

Fig. S1 The DSC curves of different hydrogels in the second heating process.

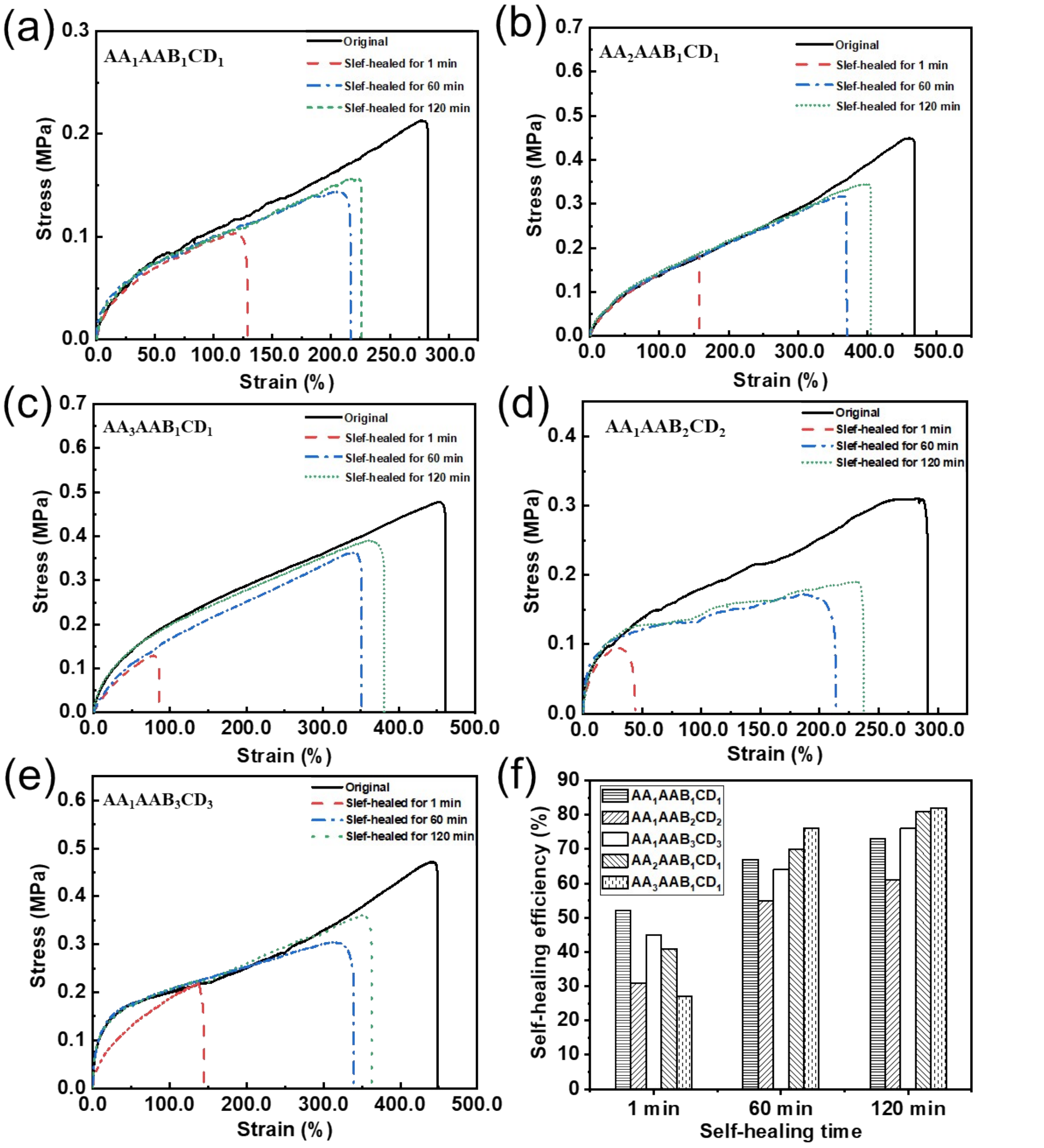

Fig. S2 The tensile strength and the self-healing efficiency of different hydrogels. The influence of AA content (a-c) and EP-CD (a, d and e) content and self-healing time on tensile strength. (f) The self-healing efficiency of corresponding hydrogel at 1, 60 and 120 min.
